# Count Rumford

**Published:** 1884-01

**Authors:** 


COUNT RUMFORD.

An article from the pen of Mr. John Tyndall, lately published in the *Contemporary Review*, recalls attention to the eventful and even romantic career of the man, who, beginning the study of medicine with Dr. John Hay, of Woburn, concluded by becoming a German nobleman and the commander of the Bavarian forces at Munich. Dr. Ellis and others have already made Americans familiar with the career of their remarkable countryman, but there is a certain charm in reading the story as narrated by an English author, who actually visited the little town of North Woburn, nine miles distant from Boston, where Rumford, or, as he was named before his elevation to the knighthood, Benjamin Thompson, first saw the light.

It is, indeed, a strange career, one scarcely possible except in those pregnant days, when Americans, then barely occupying a slender strip of the seaboard of this country, stood face to face with the nation against which they had drawn the sword. They were days when the men who determined to remove from the circumstances of early life, made excursions eastward across the sea, rather than as now to the remote regions of the West.

We read with interest of the earliest studies of the young lad in algebra, geometry, and astronomy, finding time with all this and with a weekly allowance of but two shillings and five pence, for devotion to the music of which he was passionately fond, and for perfecting his skill as a draughtsman. We trace the direction in which his mind was turned, whether in the effort to construct a "machine for perpetual motion," or to discover "the nature, essence, beginning of existence, and rise of the wind in general, with the whole theory thereof, so as to be able to answer all questions relative thereto." We are interested again in the items relating to his small economies, even crediting the doctor's wife with a pair of stockings which she knitted for him. We see him again spacing out the hours of his day: "From 11 to 6, sleep. Get up at 6 o'clock, and wash my hands and face. From 6 to 8, exercise one-half and study one-half. From 8 to 10, breakfast, attend prayers, etc. From 10 to 12, study all the time. From 12 to 1, dine. From 1 to 4, study constantly. From 4 to 5, relieve my mind by some diversion or exercise. From 5 till bed-time, follow what my inclination leads me to, whether it be to go abroad, or stay at home and read either anatomy, physic, or chemistry."

Married when but 19 years of age to a wife of 33 (the widow of Col. Rolfe), he becomes a Major in the Second Provincial Regiment of New Hampshire when less than 20. In three years more, having been twice acquitted from the taint of Toryism, we are scarcely surprised to discover him, almost immediately after his escape to Europe, officiating as under-secretary to the American Secretary of State, Inspector of Clothing, and Lieutenant Colonel Commandant of the New York Horse Dragoons. Returning to this country, we find him commanding the cavalry in South Carolina under Leslie, and in New York in active command of the King's Own. The very next year he is crossing the English channel with the historian Gibbon, who describes him as "secretary, colonel, admiral, and philosopher." Thence he pushes on to Strasburg, and is knighted by the King on the 23rd day of February, 1784. Here again we find him a colonel of a cavalry regiment, living in a palace with the Russian Ambassador, surrounded by a military staff, talking three languages, and studying the population, resources, employments, and modes of development of the people for whom he was laboring. Minister of War, Minister of Police, and Chamberlain of the Elector, he does not find it beneath his attention to plan how the soldiers, their children, and even the children of the neighboring peasantry should be taught writing, reading, and arithmetic. He drains marshes, cultivates the potato, and puts all the beggars of Bavaria into an industrial establishment. He constructs a kitchen, and provides a dinner for 2,000 people with the smallest amount of fuel which could be employed for such a purpose.

In 1796, Rumford founded the historic medal which bears his name, and soon after we find him in command of the forces of Bavaria, fighting the French and Austrians from the gates of Munich. Thence he goes with plenipotentiary powers to the court of London, and is next offered the superintendency of the Military Academy of the United States, with the office of Inspector General of the American forces.

We find him on the 13th of January, 1800, instrumental in laying the foundations of the Royal Institution of Great Britain, in conjunction with the Earl of Winchester, Lord Morton, Lord Egremont, and Sir Joseph Banks. Not the least interesting act of his singularly interesting career, is what might be called his discovery of Humphrey Davy.

This is, perhaps, a great and glorious career, but indeed not greater nor more glorious in the substantial results to the world at large than the fruitage of many lives we might name, of men who began the study of medicine in a small New England town, who were never decorated with the emblems of foreign rank, and who never led a regiment of dragoons into action.

For all this, however, such a career as Rumford's will always in one way possess special value for us. The luster which it sheds, falling on the eager eyes of many a young man whose ambitious projects might be thought to be dwarfed by straitened circumstances or a humble origin, will always point to broader enterprises and the honorable rewards of industry and well doing in small things.

